# Likely Dementia and Its Implication for Support Among the Oldest-Old in Mexico and the United States

**DOI:** 10.1093/geroni/igaa057.1907

**Published:** 2020-12-16

**Authors:** Mariana Lopez-Ortega, Silvia Mejia, Emma Aguila, Luis Gutiérrez-Robledo, William Vega, Flavia Andrade, Stephanie Grasso, Jacqueline Angel

**Affiliations:** 1 National Institute of Geriatrics, Mexico, Mexico City, Mexico; 2 El Colegio De La Frontera Norte, Tijuana, Baja California, Mexico; 3 University of Southern California, Los Angeles, California, United States; 4 The National Institute of Geriatrics, Mexico City, Distrito Federal, Mexico; 5 Florida International University, Miami, Florida, United States; 6 University of Illinois, Urbana, Illinois, United States; 7 University of Texas at Austin, Austin, Texas, United States; 8 The University of Texas at Austin, Austin, Texas, United States

## Abstract

This study examines sources of vulnerabilities to dementia in low resource populations in two specific contexts—Mexico and the United States. Data are drawn from comparable waves of the Mexican Health and Aging Study (MHAS) and the Hispanic Established Populations for the Epidemiologic Study of the Elderly (H-EPESE) in 2012, which include representative samples of the oldest-old (82 and over), the fastest growing segment of the populations worldwide. Likely dementia prevalence is 30.9% (±0.46SD) for Mexicans in Mexico and 36.3% (±0.48SD) for Mexicans in the U.S. Odds of likely dementia in both populations were increased by age, living in extended households, depressive symptoms, and Seguro Popular and Medicaid receipt. Being female and having comorbid cardiovascular conditions were also associated with likely dementia but only for older Mexicans. There is a need to strengthen the caregiving capacity of memory care services in low resource communities in Mexico and the U.S.

